# A pregnant woman with long-standing, retained intraabdominal glass shards who gave birth to a live infant with no complications: a case report

**DOI:** 10.1186/s13256-024-04392-8

**Published:** 2024-02-25

**Authors:** Kenta Inoue, Shinichiro Yabe, Soichiro Kashiwabara, Yukiko Itaya, Sumiko Era, Akihiko Kikuchi, Yasushi Takai

**Affiliations:** 1 Division of Maternal and Fetal Medicine, Center for Maternal, Fetal and Neonatal Medicine, Saitama Medical Center, Saitama Medical University, 1981 Kamoda, Kawagoe, Saitama 350-8550 Japan; 2 Department of Obstetrics and Gynecology, Saitama Medical Center, Saitama Medical University, Saitama, Japan

**Keywords:** Abdominal injuries, Foreign bodies, Glass, Penetrating wounds, Pregnancy

## Abstract

**Background:**

Most cases of traumatic injury during pregnancy involve blunt trauma, with penetrating trauma being uncommonly rare. In glass shard injuries, fragments often penetrate deeply, and multiple injuries may occur simultaneously; attention must be paid to the possibility of organ injury from the residual fragments. However, no case of this occurring during pregnancy has been reported yet.

**Case presentation:**

We present the case of a 34-year-old pregnant Cameroonian woman who retained intraabdominal glass shards following a penetrating injury at 13 weeks gestation and not diagnosed until 22 weeks gestation. Notably, this patient continued the pregnancy without complications and gave birth via cesarean section at 36 weeks gestation.

**Conclusion:**

In pregnant women sustaining a penetrating glass trauma during pregnancy, careful attention should be paid to the fragments; in that case, computed tomography is a useful modality for accurately visualizing any remaining fragments in the body. Essentially, the foreign bodies in glass shard injuries during pregnancy should be removed immediately, but conservative management for term delivery is an important choice for patients at risk for preterm delivery.

## Background

Penetrating glass traumas are extremely rare [1], and retained shards are immediately removed in most cases. There have been several case reports of small penetrating wounds wherein glass shards have remained undetected for some time, subsequently causing delayed organ injury. However, to our knowledge, no case of long-standing retention of glass fragments in pregnancy has been previously reported. This is the first report of a pregnant woman who sustained a penetrating trauma at 13 weeks gestation with several glass fragments entering the abdominal cavity, but who continued with an unremarkable pregnancy course and delivery at 36 weeks gestation. Here, we describe the modality of detecting glass shards in the body and the perinatal management.

## Case presentation

Our patient was a 34-year-old Cameroonian woman (gravida 5 para 2) who had undergone an elective cesarean section for suspected macrosomia in her home country. In light of this history, she gave birth to her second child in Japan by repeat cesarean section. She had previously undergone laparoscopic hernia repair in Japan. During a visit to her country, she was injured after falling against a glass wall, unaware of her current pregnancy. She received antibiotic treatment and blood transfusion at a local hospital, where numerous superficial glass fragments were removed from her body. Computed tomography (CT) scanning was not performed during this time. Although she became cognizant of her pregnancy afterward, she did not immediately seek consultation with an obstetrics and gynecology (OBGYN) provider. Five weeks after the accident, at 18 weeks gestation, she returned to Japan and sought consultation at her previous OBGYN clinic where she received perinatal care. At 21 weeks gestation, she presented at our hospital’s department of plastic surgery complaining of pain near the site of her hernia repair scar. Recurrence of hernia was suspected but was not evident on palpation. The physician ordered a CT scan of the abdomen.

**Fig. 1:**
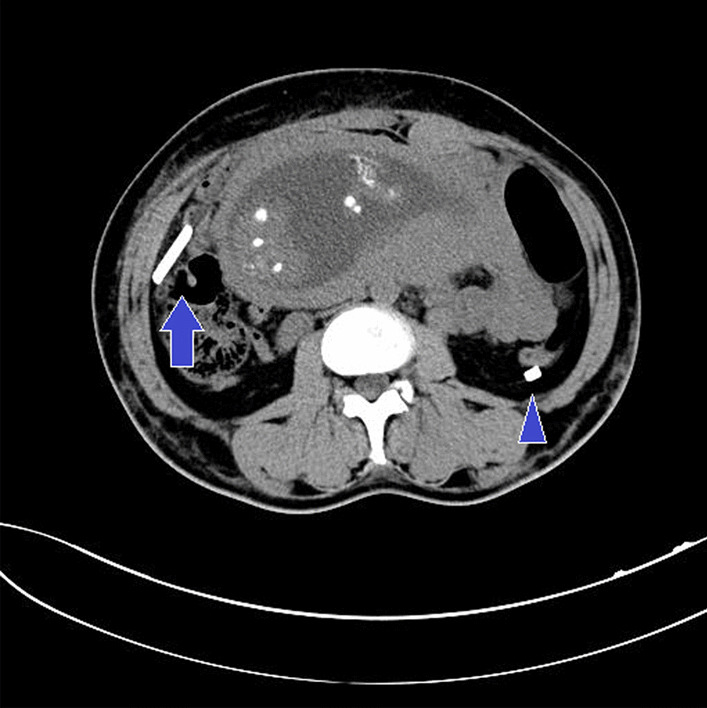
Upper abdominal plain axial CT at 22 weeks gestation. Two hyperdense foci believed to be glass shards are present in the right hypochondrium (arrow) and near the dorsal side of the descending colon (arrowhead). As the patient was pregnant, the plastic surgeon restricted the scanned area to the upper abdomen

**Fig. 2:**
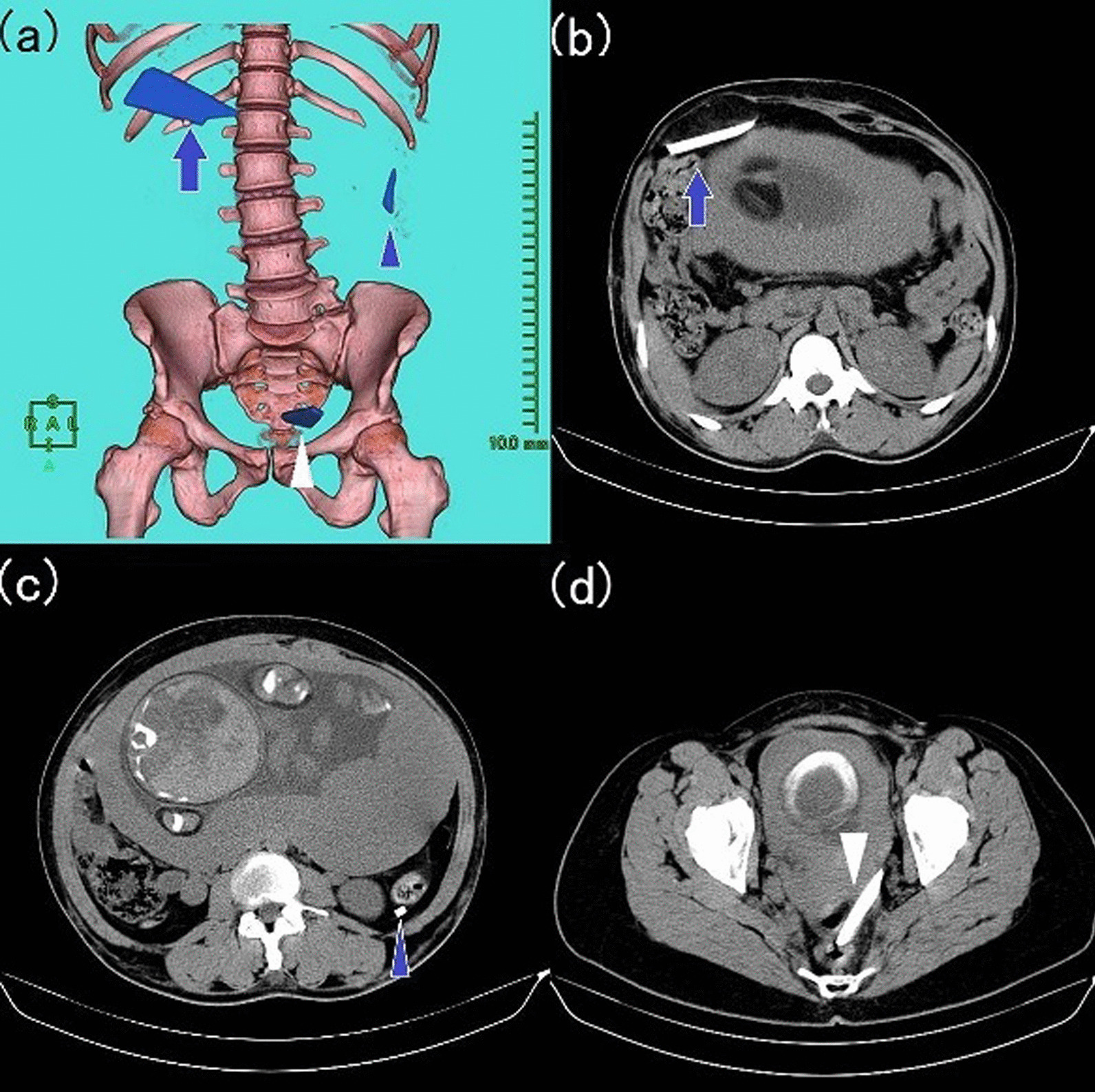
**a** Abdominopelvic three-dimensional CT and **b**-**d** axial CT images at 33 weeks gestation. The glass fragments are shown in blue on the images. There was no change in the positions of the shards in the right hypochondrium (arrow) or near the dorsal side of the descending colon (arrowhead), but the CT scan of the pelvic cavity revealed another 6 cm shard (white arrowhead) in the pouch of Douglas

Plain upper abdominal CT scan performed at 22 weeks gestation showed a 9 cm glass shard reaching the right hypochondrium and a 3 cm piece of glass shard at the dorsal side of the descending colon (Fig. 1). Her OBGYN at the time decided on conservative management with removal of fragments during cesarean section at full term, since it seemed the glass fragments were not sharp and that she was scheduled to have a cesarean section. At 27 weeks gestation, she transferred to a new residence and was referred to our hospital. After a consultation between our department and the department of emergency medicine, we concurred that the glass fragments were not sharp, and the risk of organ damage, intestinal perforation, or uterine perforation was low. It was similarly decided that the fragments would be removed during the cesarean section. The foreign bodies were reassessed by abdominopelvic CT at 33 weeks gestation (Fig. 2). The positions of the fragments in the upper abdomen were unchanged, but the scan showed another 6 cm piece of glass in the pouch of Douglas. As it was adjacent to the sigmoid colon, we again consulted the department of emergency medicine. However, removing the fragment from the pouch of Douglas would be a highly invasive operation entailing the risk of premature delivery. As no organ damage had occurred in the last 5 months since the injury, we decided to proceed with the original plan. Thus, we performed point-of-care ultrasound (POCUS) for evaluation of delayed organ damage during regular prenatal checkups. Specifically, we confirmed whether POCUS revealed the peritoneal stripe sign without sliding in the right upper quadrant for detecting pneumoperitoneum.

**Fig. 3:**
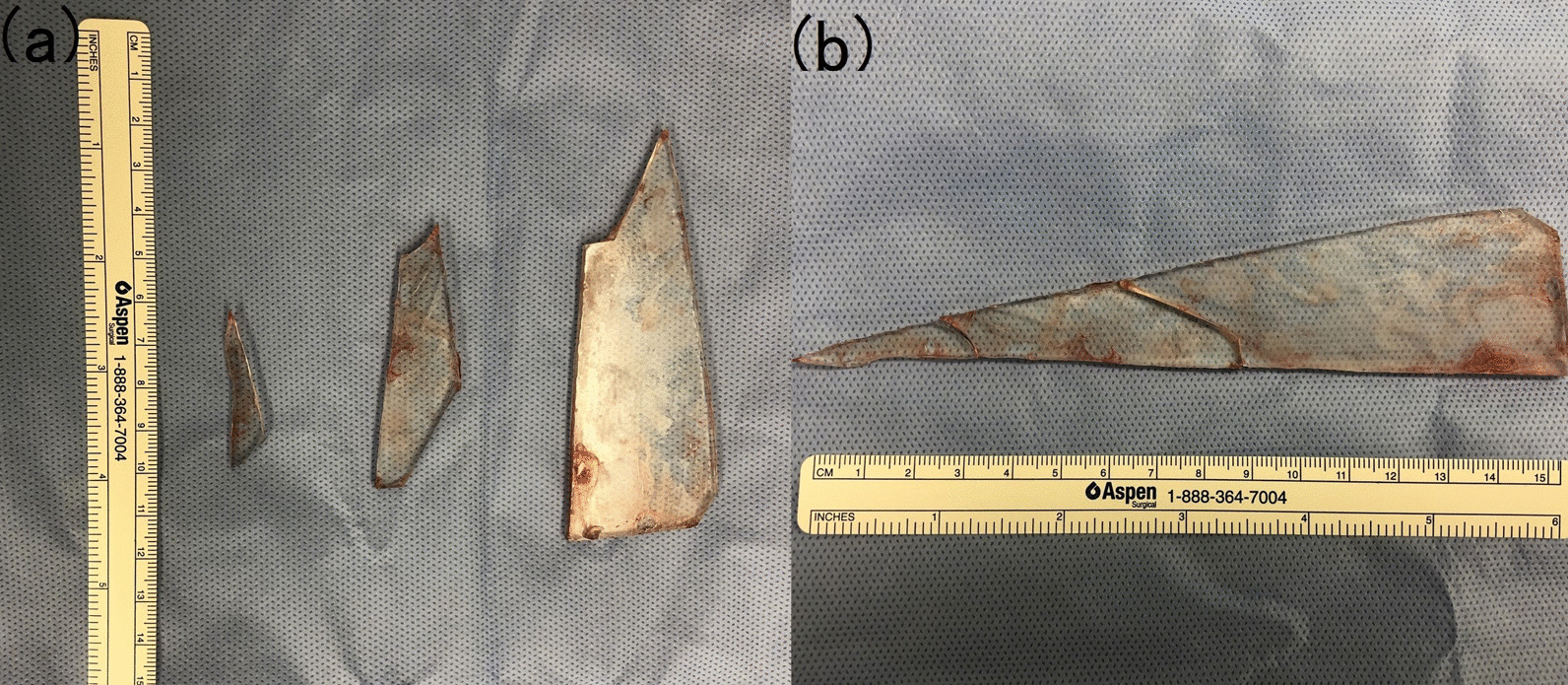
**a** The extracted glass fragments. **b** When put together, the extracted pieces were found to comprise a single shard of glass approximately 15 cm long

To avoid the possibility of emergency surgery owing to labor pain, a cesarean section was conducted at 36 weeks gestation in collaboration with the department of emergency medicine, and the glass fragments were removed. A 12-cm midline longitudinal incision was made in the lower abdomen, extending from the pubis to the umbilicus. There was no obvious scarring in or adhesions to the anterior uterine wall. A 2744-g female infant was delivered via a lower uterine segment transverse incision. After the placenta had been delivered, the uterine incision was sutured closed, and the exploration in the abdomen was initiated. The first glass fragment to be identified was found entangled in the greater omentum in the upper abdomen, and this was removed by resecting a portion of the omentum. The second glass fragment was found near the descending colon, and this was removed without adhesions. Finally, an examination of the pouch of Douglas revealed the third glass fragment with no adhesions between the fragment and the intestines; this was also removed. The abdomen was then closed after checking that there was no damage to any of the surrounding organs. The operating time was 2 hours 14 minutes, and blood loss (excluding amniotic fluid) was 535 mL. When put together, the extracted fragments of glass were found to compose a single shard (Fig. 3). Postoperative CT did not reveal any remaining glass fragments. The mother and child were both discharged uneventfully on postoperative day 6.

## Discussion and conclusion

Traumatic injury occurs in 1–8% of all pregnancies, and this has a significant impact on perinatal prognosis [2, 3]. Causes of traumatic injuries include motor vehicle accidents, falls, and violence. With most cases involving blunt trauma, the maternal prognosis after mild injury is considered to be good [3]. In contrast, a penetrating trauma is extremely rare, with one review article stating that it occurs in only 3.27 out of every 100,000 live births [4]. The maternal prognosis is reportedly poor [5], and thus, the perinatal management of such injuries is important.

In penetrating glass wounds, foreign bodies may lodge in the body and cause deep organ injury even when the superficial wound is small. Because of this, taking a detailed medical history at the time of the injury, properly confirming physical examination findings, and performing appropriate diagnostic imaging are all important.

Ultrasound is the simplest diagnostic imaging modality that may be ordered, and abdominal ultrasound is considered to be the first choice for the cases with acute abdominal pain because it is inexpensive and rapid. But its performance declines with increasing distance from the body surface [6]. In addition, given the nature of glass to form splinters, multiple shards may enter the body simultaneously, and modalities that allow a wide area to be scanned at once, such as radiography (X-ray), CT, and magnetic resonance imaging (MRI), are useful. CT reportedly outperforms X-rays and MRI in terms of detection output, sensitivity, and specificity [7]. This makes CT scanning the most suitable modality for patients with glass injuries. Note, however, that a sufficiently large area must be scanned so as to avoid missing any fragments, especially during the initial assessment. In our patient, for example, the CT scan performed during the second trimester of pregnancy did not detect the glass fragment in the pelvis because it was limited to the upper abdominal region.

In some regions, primary care clinics may not include an OBGYN department, and the providers may have psychological barriers for pregnant women to receiving a CT scan, including fear of radiation exposure. In general, the bioeffect dose of fetal radiation is more than 50 mGy, the mean fetal dose of single phase abdominopelvic CT is roughly 20 mGy [8]. In addition, attempts have been made to reduce radiation exposure during CT scans via model-based iterative reconstruction (MBIR) [9]. MBIR maintains image quality while reportedly enabling a dose reduction of 67–86% [10]. Thus there is no need to avoid CT scans for detecting glass shards.

Regarding the timing of fragment removal, there has been no previous report of residual glass shards from a penetrating trauma during pregnancy that can be used as reference. However, one review article has described a small number of cases of long-standing residual intraabdominal glass shards in nonpregnant patients [11]. In this report, the longest time a glass shard remained in the abdomen among five patients was 20 months, and three of them had delayed injury to the bowel. In our patient, two of the three glass fragments were not adherent to the surrounding tissue and might have easily migrated elsewhere. These three fragments were found to form a single shard, and it might have shattered inside the body instead, with the fragments migrating to different parts of the abdominal cavity. So, the risk of organ damage during perinatal management would be substantial. Surgery during pregnancy should be avoided whenever possible but should be performed in situations where emergency surgery is necessary [12]. In the first trimester of organogenesis, surgery should be avoided because of the effects of the drug on the fetus, and in the second trimester, the uterus is still small and the risk of preterm delivery is reported to be lower than in the third trimester [13]. Therefore, if the glass fragments had been found early in pregnancy, immediate surgical removal from the second half of the first trimester to the first half of second trimester would have been considered owing to the risk of bowel perforation.

When our patient was 33 weeks pregnant, the fragment in the pouch of Douglas could no longer be removed because of the enlarged uterus. Recently, it has been reported that ultrasonography is useful tool in diagnosis of pneumoperitoneum [14]. The procedure of detecting peritoneal stripe sign without sliding is simple [12], and we use it for assessment of gastrointestinal perforation. In the third trimester of pregnancy, the conservative management is an important choice with due regard to the risk of premature birth as a result of surgical procedures.

Regarding the delivery mode, we performed a cesarean section in this case because of a previous cesarean pregnancy. Even if she had not had a previous cesarean section, we would have chosen a cesarean section. In the present case, there was a large 6 cm piece of debris on the side of colon. As the delivery progresses, the risk of damage to the uterus was considered sufficient. If vaginal delivery is possible, the patient must then undergo laparotomy. So there is no reason to choose a vaginal delivery. If the debris had been detected early in pregnancy, laparotomy would have been considered.

Patients who sustain a penetrating glass trauma during pregnancy should undergo a CT scan of a sufficiently large area for an accurate assessment of the penetration of the glass shards. Essentially, the foreign bodies in glass shard injuries during pregnancy should be removed immediately, but conservative management for term delivery is an important choice for patients at risk of preterm delivery. Point-of-care ultrasound (POCUS) is useful for evaluation of delayed organ damage during pregnancy.

## Data Availability

Data sharing is not applicable to this article as no datasets were generated or analyzed during the current study.

## Abbreviations

CT: Computed tomography
OBGYN: Obstetrics and gynecology
POCUS: Point-of-care ultrasound

## References

[1] Greco PS, Day LJ, Pearlman MD (2019). Guidance for evaluation and management of blunt abdominal trauma in pregnancy. Obstet Gynecol.

[2] Battaloglu E, McDonnell D, Chu J (2016). Epidemiology and outcomes of pregnancy and obstetric complications in trauma in the United Kingdom. Injury.

[3] Hill CC, Pickinpaugh J (2008). Trauma and surgical emergencies in the obstetric patient. Surg Clin North Am.

[4] Mendez-Figueroa H, Dahlke JD, Vrees RA (2013). Trauma in pregnancy: an updated systematic review. Am J Obstet Gynecol.

[5] Petrone P, Talving P, Browder T (2011). Abdominal injuries in pregnancy: a 155-month study at two level 1 trauma centers. Injury.

[6] Haghnegahdar A, Shakibafard A, Khosravifard N (2016). Comparison between computed tomography and ultrasonography in detecting foreign bodies regarding their composition and depth: an in vitro study. J Dent (Shiraz).

[7] Ogden NKE, Milner PI, Stack JD (2021). CT more accurately detects foreign bodies within the equine foot than MRI or digital radiography. Vet Radiol Ultrasound.

[8] Sensakovic WF, Royall I, Hough M (2020). Fetal dosimetry at CT: a primer. Radiographics.

[9] Imai R, Miyazaki O, Horiuchi T (2017). Ultra-low-dose fetal CT with model-based iterative reconstruction: a prospective pilot study. AJR Am J Roentgenol.

[10] Miéville FA, Gudinchet F, Brunelle F (2013). Iterative reconstruction methods in two different MDCT scanners: physical metrics and 4-alternative forced-choice detectability experiments–a phantom approach. Phys Med.

[11] Luks B, Dworzyńska A, Dobrogowski M (2020). Discovery of a glass splinter in the abdominal cavity after an old impalement injury: a case report and literature review. Am J Case Rep.

[12] ACOG Committee Opinion No. 775. Nonobstetric surgery during pregnancy. Obstet Gynecol. 2019;133:e285. 10.1097/AOG.0000000000003174 30913200

[13] Faum M, Rojansky N (2001). Laparoscopic surgery during pregnancy. Obstet Gynecol Surv.

[14] Taylor MA, Merritt CH, Riddle PJ (2020). Diagnosis at gut point: rapid identification of pneumoperitoneum via point-of-care ultrasound. Ultrasound J.

